# Differentiation grade is highly concordant between matched primary and metastatic colorectal cancer

**DOI:** 10.1007/s10585-025-10380-z

**Published:** 2025-10-17

**Authors:** Camilla M. Reehorst, Jennifer K. Mooi, Charles J. Uy, Kristen A. Needham, Sarah Ellis, Ian Y. Luk, Laura J. Jenkins, Rebecca Nightingale, Fiona Chionh, Andreas Behren, Niall C. Tebbutt, David S. Williams, John M. Mariadason

**Affiliations:** 1 Olivia Newton-John Cancer Research Institute, Austin Health, 145 Studley Road, Heidelberg, VIC 3084 Australia; 2 School of Cancer Medicine, La Trobe University, Melbourne, Australia; 3 Department of Pathology, Austin Health, 145 Studley Road, Heidelberg, VIC 3084 Australia; 4 Department of Medicine, Austin Health, University of Melbourne, Melbourne, Australia

**Keywords:** Colorectal cancer, Differentiation grade, Metastasis, Epithelial to mesenchymal transition, CDX2, Mucin

## Abstract

Differentiation grade of colorectal cancers (CRC) is histologically defined by the proportion of the tumour which retains the glandular architecture of the normal colon. Poorly differentiated CRCs display loss of glandular architecture and canonical markers of colonic differentiation and are associated with increased metastatic capacity and poorer prognosis. It is currently unclear whether poorly differentiated cells within a heterogeneous primary tumour are more likely to establish metastases and if this cellular trait is conserved in the secondary tumours, or whether metastasis is largely stochastic, with most cells in the primary tumour harboring metastatic potential. To explore this, we examined the concordance in histological grade, expression of markers of colonic differentiation, and epithelial to mesenchymal transition (EMT) in 67 matched primary and metastatic CRCs. Tumour differentiation grade was scored categorically, and expression of differentiation and EMT markers were scored as continuous variables. In matched primary-metastatic pairs, tumour grade was concordant in 88% of cases (59/67 pairs), irrespective of the site of metastasis. In tumour pairs with discordant grade (n = 8), tumour grade was higher in the metastatic lesion in 6/8 (75%) cases. Consistent with the histological concordance, expression of the key driver of colonic differentiation (CDX2); colonic lineage-specific markers (VIL1, MUC2, SYN and CHG); and the markers of epithelial-to-mesenchymal transition (E-Cadherin and Vimentin) were highly concordant between matched primary and metastatic lesions. Finally, no staining of the squamous lineage marker Cytokeratin5/6 was observed in either primary or metastatic tumours. Tumour grade assessed histologically or using expression of markers of colonic differentiation or epithelial to mesenchymal transition was largely concordant between primary and metastatic CRC. These findings complement previously reported genomic similarities between primary and metastatic lesions and demonstrate that the histological grade of a primary tumour can in most cases inform the differentiation grade of associated metastatic lesions.

## Introduction

Colorectal cancer (CRC) claims the lives of over 1 million people worldwide each year. The disease is potentially curable if detected early, but is often incurable once metastasized to distant organs, where the 5-year survival rate is < 20%. Differentiation grade is a well-established prognostic factor in CRC, with poorly differentiated histology associated with more aggressive disease and enhanced metastatic propensity [1, 2].

Conventional colorectal adenocarcinomas are histologically graded as well, moderate, poorly or undifferentiated based on the abundance of glandular structures, with well differentiated CRCs comprising of > 95% glandular structures; moderately differentiated tumours comprising 50–95% glandular structures; poorly differentiated CRCs comprising < 50% glandular structures and un-differentiated tumours comprising < 5% glandular structures [3], with grading based on the least differentiated component for heterogeneous tumours [4]. Approximately 70–80% of CRCs present with well or moderately differentiated histology (low grade) [5–7], while ~ 20% of cases are poorly differentiated or undifferentiated (high grade) displaying loss of glandular structure, more solid growth patterns and reduced expression of canonical markers of the normal colon (CDX2, VIL1 and KRT20) [8–10].

The poorer prognosis of poorly differentiated CRC is likely a consequence of the increased capacity of these tumour cells to navigate the multiple processes implicated in metastasis including cell migration and local invasion, intravasation, survival in the bloodstream, extravasation and finally seeding and expansion in a new environment [11]. However, it is unclear whether subsets of poorly differentiated cells within a heterogeneous primary tumour are more likely to establish metastases, or whether metastasis is largely stochastic, with most cells in the primary tumour harbouring the capacity to metastasize. Comparison of the concordance in differentiation grade between matched primary and metastatic CRCs could shed light on this question.

Molecular profiling of primary and metastatic CRCs has demonstrated high genomic [12–16] and epigenetic [17] concordance between matched primary and metastatic lesions, enabling genomic analysis of primary tumour tissue to be used to determine eligibility of metastatic CRC patients for treatment with targeted therapies such as EGFR and BRAF inhibitors. Comparatively, immunohistochemical assessment of Ki67 in matched primary and metastatic tumours revealed that metastatic tumours display reduced proliferation rates [18]. A recent single cell profiling study by Moorman et al. also found that metastasized colorectal cancer cells in some cases transitioned into squamous and neuroendocrine-like cell states, which was associated with poorer clinical outcomes [19]. However, the concordance in histological grade between matched primary and metastatic colorectal cancers has not been systematically addressed, a question of clinical significance given the association between poorly differentiated histology and poorer patient outcome.

In this study we report that differentiation grade, and expression of differentiation and EMT markers, are highly concordant between matched primary and metastatic CRCs irrespective of the site of metastasis, and therefore that histological grade of a primary tumour can reliably predict the differentiation grade of associated metastatic lesions.

## Methods

### Patient cohorts and histological grading

Matched formalin-fixed paraffin-embedded (FFPE) primary and metastatic colorectal tumour samples were obtained from 67 patients who underwent surgical resection of their primary and metastatic tumour at Austin Health in Melbourne (n = 29), or who participated in the Phase III MAX clinical trial (n = 38) [20]. The metastases comprised both synchronous and metachronous cases. Only a single matched metastasis was available for study for each primary tumour and all metastatic tumour samples were from surgical resections with sufficient material for tissue microarray generation. In the MAX cohort, 11 of the 38 patients received prior adjuvant chemotherapy or radiotherapy (7 chemotherapy alone, 1 radiotherapy alone and 3 chemoradiotherapy). Prior adjuvant chemotherapy data was not available for the Austin Health cohort. Patients participating in the MAX trial provided informed consent at the time of enrolment, while samples from the Austin Health archives were obtained using an Austin Health HREC approved waiver of consent (H2004/01986). Assembly of tissue microarrays of these cases has been described previously, [18, 21] with each tumour and metastatic sample represented by three cores taken from different regions of the tumour. Differentiation grade was scored on full face tumour sections by an Anatomical pathologist (DW) using the criteria outlined in the 2019 WHO Classification of Tumours 5th Edition (Digestive System Tumours) [4, 22]. For heterogeneous tumours, grade was based on the least differentiated component. As the minimum area of high-grade tumour required for this classification has not been defined, we applied the criteria that if there was a discrete low power field (4 × objective) of high-grade tumour present within a heterogeneous tumour, the tumour was scored as high grade.

### Immunohistochemistry

Immunohistochemistry was performed on 4-micron sections of TMAs. Deparaffinization and rehydration, antigen retrieval and primary antibody incubation was performed at conditions specified in Table 1. The secondary antibody and detection system for automated IHC was either the Ventana OptiView or ultraView Universal DAB Detection Kit using the Ventana BenchMark Ultra platform (Ventana Medical Systems, Tucson, AZ, USA). All sections were then counterstained with Mayer’s haematoxylin.

**Table 1:** Immunohistochemistry staining conditions

Antibody name	Method	Antigen retrieval method	Primary Ab incubation	Dilutions	Supplier	Clone
CDX2	Automated	EDTA pH 8, 98 °C, 36 min	36 °C, 32 min	50	Dako	DAK-CDX2
Villin (VIL1)	Automated	EDTA pH 8, 95 °C, 36 min	36 °C, 32 min	50	Novocastra	CWWB1
MUC2	Automated	EDTA pH 8, 95 °C, 52 min	36 °C, 32 min	50	Dako	CCP58
Chromogranin (CHG)	Automated	EDTA pH 8, 100 °C, 8 min	36 °C, 8 min	RTU	Ventana	LK2H10
Synaptophysin (SYN)	Automated	EDTA pH 8, 100 °C, 32 min	36 °C, 32 min	RTU	Ventana	SP11
E-Cadherin	Automated	EDTA pH 8, 95 °C, 52 min	36 °C, 8 min	RTU	Ventana	EP700Y
Vimentin	Automated	EDTA pH 8, 100 °C, 32 min	36 °C, 16 min	RTU	Ventana	V9
Cytokeratin 5/6	Automated	EDTA pH 8, 100 °C, 32 min	36 °C, 16 min	RTU	Ventana	D5/16B4

RTU (ready to use)

### Scoring of IHC markers

VIL1, MUC2, CHG, SYN, E-Cadherin and Vimentin were manually scored according to the criteria outlined below (Table 2). Nuclear CDX2 staining and total MUC2 was scored using the HALO/AI™ Image Analysis Software (Indica Labs, New Mexico, USA). Staining of each marker was highly homogeneous across all three cores for all tumours.

**Table 2:** Scoring criteria for IHC markers

IHC marker	Scoring criteria
Villin	Cell membrane staining of tumour cells: 0—absent 1—weak, and/or < 70% of tumour cells stained positive 2—moderate, and > 70% of tumour cells stained positive 3—strong and > 70% of tumour cells stained positive
CDX2	Staining intensity was determined by computation of the nuclear staining H-score using the HALO software
MUC2	Staining intensity determined by computation of H-score using the HALO software
CHG	Pos—> 1% of tumour cells positive Neg—< 1% of tumour cells positive
SYN	Pos—> 1% of tumour cells positive Neg—< 1% of tumour cells positive
E-Cadherin	Cell membrane or cytoplasmic staining of tumour cells: 0—absent 1—weak, and/or < 70% of tumour cells stained positive 2—moderate, and > 70% of tumour cells stained positive 3—strong and > 70% of tumour cells stained positive
Vimentin	Any tumour staining 0—absent 1—weak, and/or < 70% of tumour cells stained positive 2—weak –moderate, and > 70% of tumour cells stained positive 3—strong and > 70% of tumour cells stained positive
Cytokeratin 5/6	Any tumour staining 0—absent 1—weak, and/or < 70% of tumour cells stained positive 2—weak –moderate, and > 70% of tumour cells stained positive 3—strong and > 70% of tumour cells stained positive

### Multiplex immunohistochemistry staining

Opal multiplex immunohistochemistry (mIHC) was used to simultaneously detect expression of E-Cadherin, Vimentin, and pan-cytokeratin. TMAs were baked overnight at 60 °C and the following day, were subjected to deparaffinization and rehydration with xylene, graded ethanol (100% and 70% v/v), and finally dH_2_O. Sections were then subjected to heat-induced epitope retrieval (HIER) by submerging the slides in pH9 or pH6 antigen retrieval (AR) buffer (Akoya Biosciences, AR900250ML and AR600250ML, Akoya Biosciences) and microwaving at 98 °C (20% power) for 15 min. Slides were washed with Tris-buffered saline with 0.1% Tween® 20 detergent (TBST) then incubated with BLOXALL® (Vector Laboratories) for 10 min to inactivate endogenous peroxidases and alkaline phosphatases in the tissue. Blocking/Antibody diluent (ARD1001EA, Akoya Biosciences) was added to the slides for 20 min at room temperature in a humid chamber. The blocking buffer was then removed and the sections incubated with anti-E-cadherin primary antibody (BD Biosciences, Clone 36, 1:1000, for 30 min at RT) in a humidity chamber. The primary antibody was then removed and the slide washed 3X in TBST for 2 min each. Sections were subsequently stained with HRP-conjugated secondary antibody (MP-7500, Vector Laboratories) for 30 min at RT in a humidity chamber. The secondary antibody was then removed and the section washed in 2X TBST for 2 min each. To generate the fluorescent signal, an Opal fluorophore conjugated to tyramide molecules was diluted using 1X Plus Amplification Diluent (FP1498, Akoya Biosciences) and added to the slides for 10 min at RT in a humid chamber. This procedure was then repeated from HIER to addition of Opal fluorophore to stain sections with anti-vimentin (Abcam, Clone V9, 1:1000, RT, 30 min) and anti-panCK (AKOYA, OP7TL400TDS, 1:2000, RT, 30 min) antibodies. Once all markers had been stained, the slides were subjected to a final round of HIER and stained with DAPI solution (1 drop diluted in 500 μl TBST) for 5 min. After a final TBST and dH_2_O wash the sections were mounted with a glass coverslip using a hard-setting mounting media (H-1700, Vector Laboratories). Slides are left to dry overnight at 4 °C in the dark before images were taken.

### Multispectral image acquisition

Multispectral images of the stained slides were captured using the VECTRA 3.0® Automated Multispectral Imaging System (Akoya Biosciences), using the instrument’s five fluorescent filter cubes, DAPI, FITC, Cy3, CY5, and Texas Red. Two sets of exposure times (one for whole slide scans at 10 × magnification, and one for multispectral images at 20x) were individually set for each slide using each filter. Multispectral images were then spectrally unmixed using inForm® Tissue Analysis Software (Akoya Biosciences) using a custom spectral library. The images were then exported as component Tiffs for analysis using HALO/AI™ (Indica Labs) Image Analysis Software. Using the Highplex Fluorescence module, the AI was first trained to classify the tumour region (PanCK +), the stromal region, and finally the non-tissue region (no fluorescent signal). Tumour cells expressing pan-cytokeratin and E-cadherin, but not vimentin, were categorized as “epithelial”; tumour cells expressing pan-cytokeratin and both E-Cadherin and vimentin were categorized as “transitioning”; and tumour cells expressing pan-cytokeratin and vimentin but not E-cadherin were categorized as “mesenchymal” using the software’s phenotype function. At least 1000 pan-cytokeratin positive cells were classified per core.

## Results

### Differentiation grade of matched primary and metastatic CRC is highly concordant.

To determine the concordance in histological grade between primary and metastatic CRCs, 67 matched primary and metastatic CRCs were assembled and histologically graded.

**Fig. 1:**
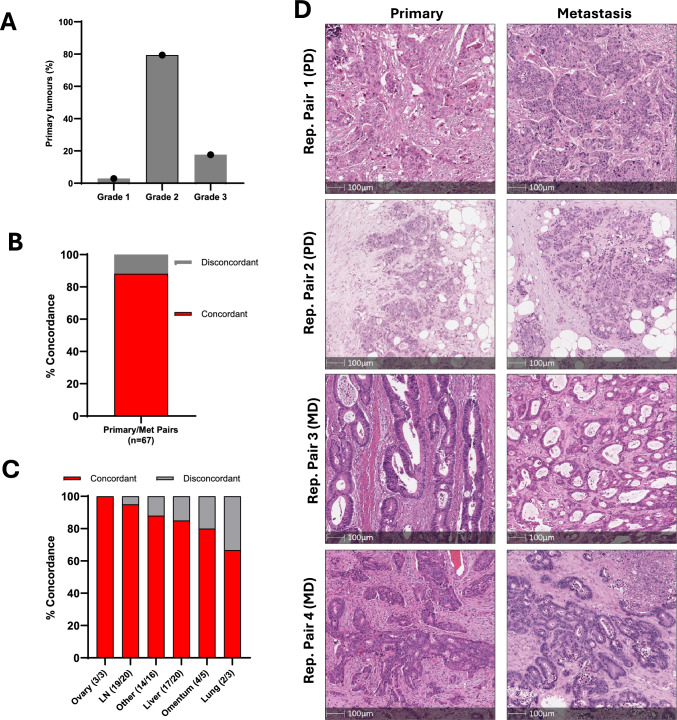
Matched primary and metastatic CRCs show a high degree of concordance in differentiation grade. **A** Differentiation grade of primary tumours (n = 67). **B** Overall concordance in histological grade between matched primary and metastatic CRCs. **C** Concordance in histological grade of matched primary and matched metastases according to metastatic site. **D** Representative H&E images of matched primary and metastatic CRCs (PD: Poorly differentiated; MD: Moderately differentiated)

Of the primary tumours, n = 2 (2.9%) were well differentiated, n = 54 (79.4%) moderately differentiated, and n = 12 (17.6%) poorly differentiated (Fig. 1A). This was similar to previously reported frequencies of tumour grade for CRC, with the exception that the percentage of well-differentiated tumours (2.9%) was lower than the ~ 10% reported in previous studies [23], which likely reflects this being a metastatic cohort. The overall rate of concordance in histological grade between matched primary and metastatic CRCs was 88.1% (59/67 cases) (Fig. 1B). While sample sizes for some anatomical locations were small, the rate of concordance in histological grade was highest for tumours which had metastasized to the ovaries (100%, 3/3) and lymph nodes (19/20, 95%), and slightly lower for tumours which had metastasized to the liver (17/20, 85%), omentum (4/5, 80%) and lung (2/3, 66.7%)(Fig. 1C, D). In tumour pairs with discordant grade (n = 8), most metastatic tumours (6/8, 75%) were higher grade (less differentiated) than primary tumours. In the subset of cases for whom prior adjuvant chemotherapy or radiotherapy was available (n = 38), the discordance rate was higher in those who received chemotherapy (3/11, 27%, higher grade in the metastasis in all 3 cases) compared to those who did not receive chemotherapy or radiotherapy (3/27, 11%).

### Expression of the colonic epithelial lineage driver CDX2 and colonic lineage marker VIL1 is highly concordant between matched primary and metastatic CRCs

To independently validate the histological concordance observed between matched primary and metastatic CRCs, we determined expression of the colonic lineage-specific transcription factor CDX2 and the differentiation marker Villin (VIL1), in the matched primary and metastatic lesions by IHC. For CDX2, staining intensity and H-score was assessed using the HALO image analysis software (methods), while membrane staining of VIL1 was manually scored.

**Fig. 2:**
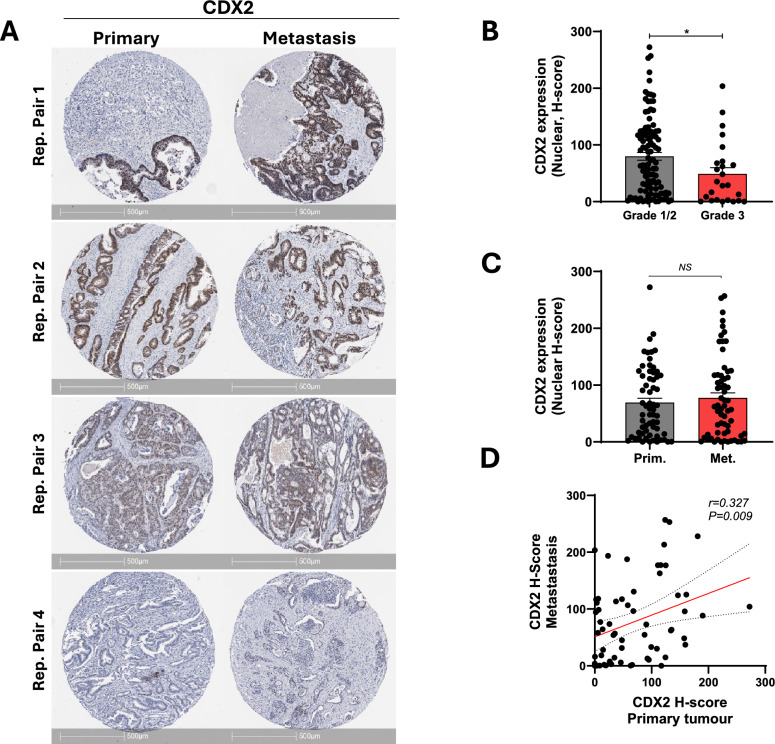
CDX2 is concordantly expressed between primary and matched metastatic CRCs. **A** Representative images of CDX2 expression in matched primary and metastatic CRCs. Representative tumour pairs with high and low CDX2 expression levels are shown. **B** CDX2 staining intensity in low grade (Grade 1/2) and high-grade (Grade 3) CRCs (primary and metastatic cases). **C** Summary of CDX2 expression (H-score) in matched primary and metastatic cases (n = 63 pairs, NS: not significant, paired students t-test). **D** Pearson’s correlation of CDX2 expression levels (H-score) between matched primary and metastatic CRCs (n = 63 pairs)

Analysis of CDX2 expression across the cohort of primary and metastatic tumours demonstrated a range of nuclear expression levels (Fig. 2A), with significantly higher expression in well/moderately differentiated (low grade) compared to poorly differentiated (high grade) tumours (Fig. 2B) as reported previously [8, 24, 25]. Comparatively, we found no difference in the overall level of CDX2 expression between matched primary and metastatic tumours (Fig. 2C), as well as a significant positive correlation in CDX2 expression levels between individual matched primary and metastatic cases (Fig. 2D), collectively indicating concordant CDX2 expression between matched primary and metastatic lesions.

**Fig. 3:**
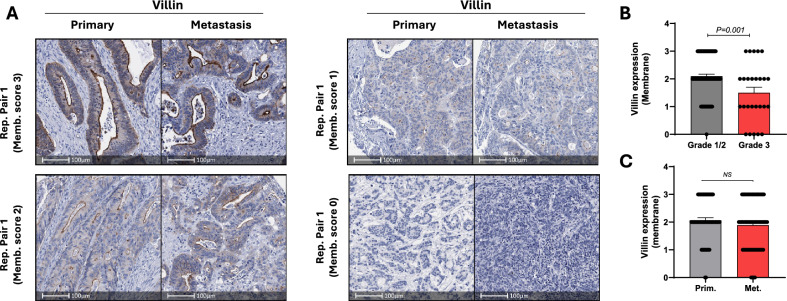
Villin is concordantly expressed between primary and matched metastatic CRCs. **A** Representative images of Villin (membrane) expression in matched primary and metastatic CRCs. Representative primary and matched metastatic tumour pairs with membrane staining intensities of 3, 2, 1 and 0 are shown. **B** Villin membrane expression in low grade (Grade 1/2) and high-grade (Grade 3) CRCs (primary and metastatic cases). **C** Summary of Villin membrane expression in matched primary and metastatic cases (n = 55, NS: not significant, paired students t-test)

We next assessed expression levels of the brush border protein VIL1, which as expected, was highest in the apical membrane of tumour cells (Fig. 3A). Consistent with our previous findings, membrane expression of VIL1 expression was significantly higher in well/moderately differentiated (low grade) compared to poorly differentiated (high grade) tumours (Fig. 3B). Comparatively, as observed for CDX2, analysis of membrane VIL1 staining in 55 matched pairs revealed similar expression between primary and matched metastases (Fig. 3C).

### Mucinous histology and expression of the goblet cell marker MUC2 are highly concordant between matched primary and metastatic CRC

**Fig. 4:**
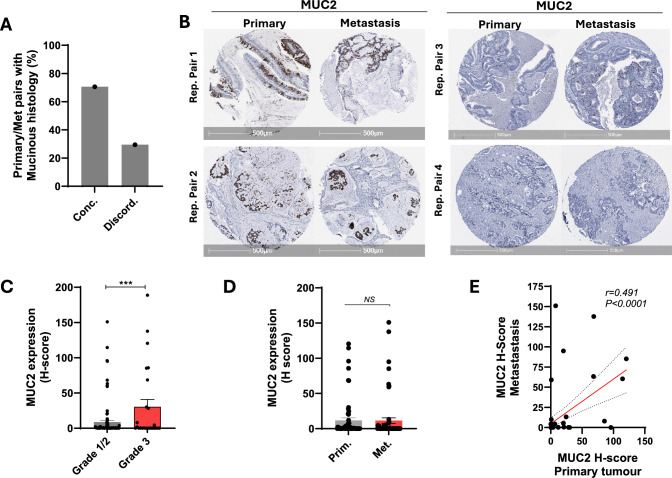
Mucinous histology and MUC2 expression are concordant between primary and matched metastatic CRCs. **A** Percentage of matched primary and metastatic CRCs with concordant (conc.) and discordant (discord.) mucinous histology (n = 62). **B** Representative images of MUC2 expression in matched primary and metastatic CRCs. Representative tumour pairs with high and low MUC2 expression levels are shown. **C** MUC2 expression in low grade (Grade 1/2) and high-grade (Grade 3) CRCs (primary and metastatic tumours). **D** Summary of MUC2 expression (H-score) in matched primary and metastatic cases (n = 62, NS: not significant, paired students t-test). **E** Pearson’s correlation of MUC2 expression levels (H-score) between matched primary and metastatic CRCs (n = 55 pairs)

Next, we examined the rate of concordance in features of mucinous histology between primary and matched metastatic tumours. Histopathological assessment of mucinous features in the 67 matched pairs, identified 17 pairs with mucinous features in either the primary or metastatic sample, of which 12 (70.6%) displayed concordance between the primary and metastatic site (Fig. 4A). To validate these findings, all tumours were immunohistochemically stained for the goblet cell marker MUC2 and the H-score computed using the HALO image analysis software (Fig. 4B). MUC2 expression was significantly higher in cores histologically classified as mucinous, compared to non-mucinous, and MUC2 expression was significantly higher in high grade tumours (Fig. 4C). As observed for CDX2 and Villin, expression of MUC2 was similar in matched primary and metastatic lesions (Fig. 4D), and a positive statistically significant correlation in MUC2 staining intensity was observed between matched primary and metastatic cases (r = 0.49, *P* < *0.001*) (Fig. 4E).

### Expression of neuroendocrine markers is uncommon in primary and metastatic CRC

**Fig. 5:**
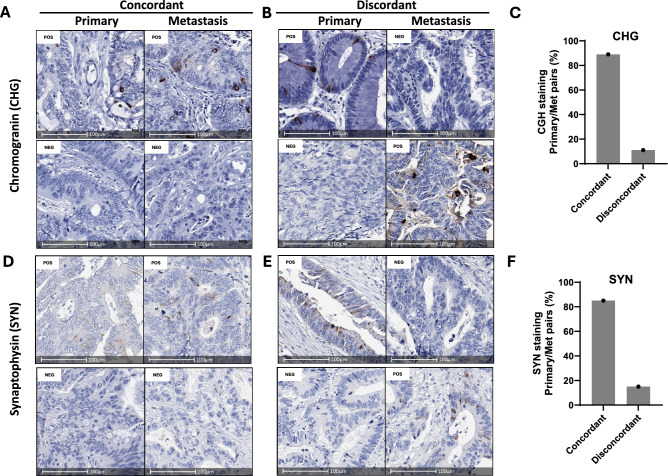
Matched primary and metastatic CRCs show a high degree of concordance in expression of neuroendocrine lineage markers. **A** Representative images of matched primary and metastatic CRCs with positive (top panel) or negative chromogranin (CHG) staining. **B** Matched primary and metastatic CRCs with discordant CHG staining. **C** Rate of concordant and discordant CHG staining between matched primary and metastatic CRCs. **D** Representative images of matched primary and metastatic CRCs with positive (top panel) or negative (bottom panel) synaptophysin (SYN) staining. **E** Matched primary and metastatic CRCs with discordant Synaptophysin staining. **F** Rate of concordant and discordant SYN staining between matched primary and metastatic CRCs

Next, we determined expression of the neuroendocrine lineage markers Chromogranin (CHG) and Synaptophysin (SYN) in matched primary and metastatic cases. Due to limited tissue availability, this analysis was only performed on 29 pairs (Austin Health cohort). No staining for CHG or SYN was observed in either the primary or matched metastasis in 82% and 69% of pairs respectively, with rare positive cells (1–10% of tumour cells) observed in the remaining cases (Fig. 5A, D). The overall rate of concordance in staining between primary and matched metastases (including negatively stained samples) was 89% for CHG (Fig. 5C) and 85% for SYN (Fig. 5F). Of the 3 cases discordant for CHG staining (Fig. 5B), positive staining in only the metastasis was observed in 2 cases and positive staining only in the primary observed in the other case. Comparatively, of the 4 cases discordant for SYN staining (Fig. 5E), positive staining in only the metastasis was observed in 3 cases, while positive staining only in the primary was observed in 1 case. Overall, therefore, while positive staining for neuroendocrine markers was relatively rare in CRCs, the staining pattern was mostly concordant between primary and matched metastases.

### Expression of epithelial and mesenchymal markers is highly concordant between matched primary and metastatic CRC

**Fig. 6:**
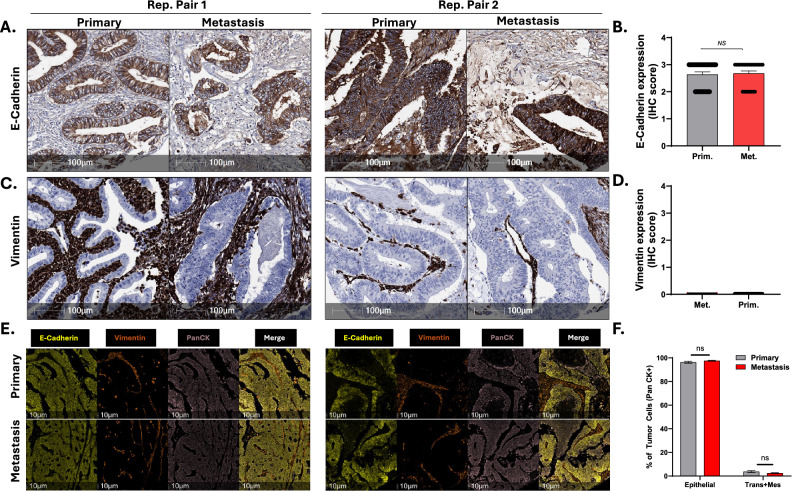
Matched primary and metastatic CRCs show a high degree of concordance in expression of E-Cadherin and Vimentin. **A** Representative images of E-Cadherin staining in 2 matched primary and metastatic CRCs. **B** Quantitation of E-Cadherin staining intensity in matched primary and metastatic CRCs (n = 28). **C** Representative images of Vimentin staining in 2 matched primary and metastatic CRCs. **D** Quantitation of Vimentin staining intensity in matched primary and metastatic CRCs (n = 28). **E** Representative matched primary and metastatic CRCs co-stained with E-Cadherin, Vimentin or pan-cytokeratin, and merged images. **F** Proportion of primary and metastatic tumour cells classified as “Epithelial” or Mesenchymal based on overlap of staining of E-Cadherin and pan-cytokeratin (epithelial) or Vimentin and pan-cytokeratin (mesenchymal)

Next, to determine if primary and metastatic CRCs display altered expression of epithelial and mesenchymal markers, we investigated expression levels of the epithelial marker E-Cadherin and the mesenchymal marker Vimentin in 29 of the matched primary and metastatic cases (Austin Health cohort). Tumour cells in all primary and metastatic cases stained positively for E-cadherin (score >  = 2, methods), and quantitation of staining intensity revealed similar levels between primary and matched metastases (Fig. 6A, B). Comparatively, while strong stromal staining for vimentin was observed in all cases, no positive staining of tumour cells (score > 0) was observed in any of the primary or metastatic cases (Fig. 6C, D).

To independently validate this analysis, we used multispectral imaging in which the same section was co stained with E-Cadherin and Vimentin as well as pan-cytokeratin to highlight epithelial tumour cells (Fig. 6E), and tumour cells classified as epithelial, transitioning or mesenchymal based on the staining patterns (methods). In both primary and matched metastases, the majority of pan-CK positive cells were classified as epithelial (96.3% vs 97.6% for primary and metastatic tumours respectively), which was not statistically different (*P* > 0.05) (Fig. 6F). The remainder of the cells were classified as either transitioning or mesenchymal (3.7% in primary vs 2.5% in metastases) which was also not statistically significant (*P* > 0.05).

### The non-canonical squamous cell marker CK5/6 is minimally expressed in primary and matched metastatic CRC lesions.

**Fig. 7:**
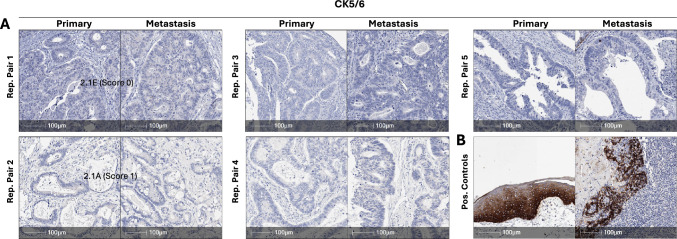
Matched primary and metastatic CRCs show minimal expression of the squamous lineage marker CK5/6. **A** Representative images of CK5/6 staining in matched primary and metastatic CRCs. All cases showed negative staining. **B** Positive staining for CK5/6 in squamous epithelium (left) and tonsil (right) shown as a positive control

A recent single cell RNA-seq profiling study reported that the expression of non-canonical lineage markers including squamous, neuroendocrine and mesenchymal lineage markers are enriched in metastatic CRC lesions [19], however other single cell profiling studies have failed to confirm this [26]. To address this in our cohort of matched primary and metastatic cases, we examined expression of the squamous lineage marker, Cytokeratin 5/6. All primary and matched metastases stained negative for CK5/6, which nevertheless indicated concordant staining of this marker (Fig. 7A).

## Discussion

Previous studies have shown that the genomic and epigenetic aberrations observed in metastatic colorectal cancers are highly concordant with those observed in matched primary samples [12–15, 17]. Comparatively, the concordance of differentiation grade between matched primary and metastatic lesions is unknown. By histopathological analysis of 67 matched primary and metastatic CRCs, we observed a high rate of concordance (88%) in differentiation grade of primary and metastatic lesions from the same patient. This was further supported by similar expression levels of general colonic and lineage-specific differentiation markers (CDX2, VIL1, MUC2, CHG and SYN) and markers of epithelial-to-mesenchymal transition (E-Cadherin, VIM). These findings corroborate that of a previous study in which expression of the differentiation marker GPA33 was found to be highly concordant between 6 pairs of primary CRCs and matched liver metastases [27].

The findings reported herein has implications for our understanding of the mechanisms driving metastatic spread. First, it suggests that in most cases the metastatic process in CRC is unlikely to be driven by permanently undifferentiated subclones within a heterogenous primary tumour. Nevertheless, given our finding that a small subset of cases display discordance in differentiation grade, it remains possible that in some cases metastasis is driven by subclones within the primary tumour, or that tumour cells undergo permanent changes in differentiation during metastasis. We also emphasize that the overall concordance in differentiation grade between primary and matched metastases is not at odds with plasticity models in which tumour cells reversibly undergo processes such as hybrid epithelial-to-mesenchymal transition during the metastatic process and revert to their inherent phenotype upon colonization at a distant site [11].

Our observation that tumour differentiation grade remains largely preserved despite the extensive physical and microenvironmental exposures cells experience during metastasis, is also consistent with the finding that histopathological and molecular features of high and low-grade CRC are preserved when tumours are grown as cell lines, patient-derived tumour organoids, or as patient-derived xenografts, involving growth in artificial conditions for extended periods [28, 29]. These findings suggest that the molecular determinants of differentiation status are robustly programmed within a tumour. This is further supported by studies illustrating that oncogenic events such as *BRAF* mutations are strongly associated with high tumour grade [30], and our recent study demonstrating that several markers of colonic differentiation which are suppressed in high grade CRCs are associated with promoter methylation [28].

Given the association of high tumour grade with poorer outcome [1], our findings indicate that patients with metastases derived from high grade primary tumours may continue to require more aggressive treatment as currently occurs in stage II disease where patients with high tumour grade are recommended to receive adjuvant chemotherapy [31]. Additionally, while there are currently no treatments tailored to the differentiation grade of CRCs; should such treatments emerge, or differentiation grade serve as a predictive biomarker of treatment response, the histology of the primary tumour may be used to inform treatment of resulting metastases. In this regard, previous studies have linked histological features with treatment response including poorer responses of NSCLCs with gastrointestinal / mucinous features to KRAS G12C inhibitors [32].

Our study nevertheless has some limitations. First, the sample size, while reasonable, was only 67, and some markers could only be assessed in a subset of these cases due to limited tissue availability. Validation of these findings in larger cohorts would therefore be of value. Second, the number of well-differentiated cases in the cohort was low, although this likely reflects the low proportion of well-differentiated CRCs overall (10–20%), and the likelihood that they will be further underrepresented in metastatic studies given their inherently better prognosis [1, 2]. Third, the techniques used to assess differentiation grade were histology and protein-based, which lack the resolution and breadth of techniques such as single cell sequencing. Nevertheless, a previous multi-omics study of a small number of cases reported that expression of EMT markers (KRT8, 18, 19, 20, CLDN 3, 4, 7, VIM) and driver genes (TWIST1, 2; SNAI1, 2, ZEB1) are largely concordant between matched primary and metastatic cases [17]. Similarly, a recent single cell profiling study revealed that the transcriptome of liver metastases, including the prevalence of different cell states (differentiated colonic lineages, transit-amplifying cells, stem cell subsets) was highly similar to that of primary tumours [26].

Notably, other studies have suggested that lymph node and distant metastases arise from independent subclones within the primary tumour [11, 33], and that lymph node metastases display greater genetic diversity / polyclonality compared to distant metastases suggesting they develop through distinct evolutionary mechanisms [34]. A study by Moorman et al. also reported increased plasticity and expression of non-canonical lineage markers in metastases compared to matched primary tumours. This was not observed in our study, however only 1 non-canonical marker (CK5/6 was assessed). Notably, in the Moorman study these changes were more pronounced in liver metastases that had received chemotherapy [19]. In our study, receipt of adjuvant chemotherapy or radiotherapy was confirmed in 11 matched cases, which notably displayed a higher rate of discordance in differentiation grade (27%) compared to those who did not receive chemo/radiation therapy (11%). We note however that sample sizes are small, and these findings need to be validated in studies specifically designed to address this question including controlling for the type of treatment received, and the time between receipt of therapy and recurrence of metastatic disease.

In summary, our analysis of primary and matched colorectal cancer metastases revealed strong concordance in differentiation grade. These findings enhance our understanding of the metastatic process, can inform the development of strategies aimed at inhibiting metastases, and potentially for informing treatment response.

## Data Availability

No datasets were generated or analysed during the current study.
